# Range of glucose as a glycemic variability and 3–month outcome in diabetic patients with acute ischemic stroke

**DOI:** 10.1371/journal.pone.0183894

**Published:** 2017-09-07

**Authors:** Young Seo Kim, Chulho Kim, Keun-Hwa Jung, Hyung-Min Kwon, Sung Hyuk Heo, Beom Joon Kim, Young Dae Kim, Jeong-Min Kim, Seung-Hoon Lee

**Affiliations:** 1 Department of Neurology, Hanyang University School of Medicine, Seoul, Republic of Korea; 2 Department of Neurology, Chuncheon Sacred Heart Hospital, Chuncheon, Republic of Korea; 3 Department of Neurology, Seoul National University Hospital, Seoul, Republic of Korea; 4 Department of Neurology, Seoul National University Seoul Metropolitan Government Boramae Medical Center, Seoul, Republic of Korea; 5 Department of Neurology, Kyung Hee University School of Medicine, Seoul, Republic of Korea; 6 Department of Neurology and Cerebrovascular Center, Seoul National University Bundang Hospital, Seongnam, Republic of Korea; 7 Department of Neurology, Severance Hospital, Yonsei University College of Medicine, Seoul, Republic of Korea; 8 Department of Neurology, Chung-Ang University Hospital, Seoul, Republic of Korea; Massachusetts General Hospital, UNITED STATES

## Abstract

Glycemic variability (GV) is reportedly a predictor for poor outcome in various clinical conditions. We aimed to assess whether GV during hospital admission is associated with poor outcomes in patients with acute ischemic stroke (AIS) and diabetes. We prospectively enrolled consecutive patients with AIS from the registry of 6 tertiary hospitals between January 2013 and December 2014. For the GV index, we used a glucose level range that was divided into 4 quartiles. Multivariable binary and ordinal logistic regression analyses were performed to determine the association between GV and the modified Rankin Scale score (3–6) at 3 months. We enrolled 1,504 patients with AIS and diabetes (mean age, 68.1 years; male, 57.2%), of which 35.1% had poor outcomes at 3 months. An increasing glucose range quartile was positively associated with initial neurologic severity and development of hypoglycemia during hospital admission. Multivariable analysis showed that the glucose level range quartile was associated with poor outcomes, even after adjusting for the number of glucose measurement and hypoglycemia (odds ratio [OR] Q2 vs. Q1: 1.50, 95% confidence interval [CI]: 1.02–2.18; OR Q3 vs. Q1: 2.01, 95% CI: 1.34–3.01; OR Q4 vs. Q1: 1.98, 95% CI: 1.22–3.23). These associations remained significant after dichotomization according to glycated hemoglobin levels at admission. An increasing glucose level range as a GV index during hospital admission was associated with poor functional outcomes at 3 months in patients with AIS and diabetes.

## Introduction

Diabetes is associated with increased risks and worse prognosis in cases with acute ischemic stroke (AIS) [1,2]. Patients with hyperglycemia at admission and during hospitalization, which is primarily noted among diabetic patients, are more likely to have worse clinical outcomes as compared to those with normoglycemia [3–5]. Hyperglycemia has a deleterious effect in cases of cerebral ischemia due to increased oxidative stress [6], inflammation [7], apoptosis [8], and inhibition of fibrinolysis [9]. However, the beneficial effects of intensive glucose lowering on the clinical outcomes after AIS remain unclear.

In the Diabetes Control and Complications Trial, a reduction in the glycated hemoglobin (A1c) levels was associated with a decreased incidence and progression of microvascular complications in patients with diabetes [10]. However, the observation of a higher risk of retinopathy progression in the conventional treatment group than in the intensive treatment group with similar A1c levels suggests that glycemic variability (GV) could potentially be linked to the development of diabetic complications [11]. Increased fluctuation of blood glucose levels is reported to be associated with poor outcomes in critically ill patients [12,13]. Although several studies have suggested that the nervous system is vulnerable to glycemic excursion [14,15], the role of GV in the outcomes of patients with AIS remains unclear. Therefore, we hypothesized that high GV would be associated with poor functional outcomes in patients with AIS, irrespective of the presence of hyper- or hypoglycemia. In the present study, we aimed to evaluate the association between GV and functional prognosis in patients with diabetes after AIS.

## Materials and methods

### Study design and participants

**Fig 1:**
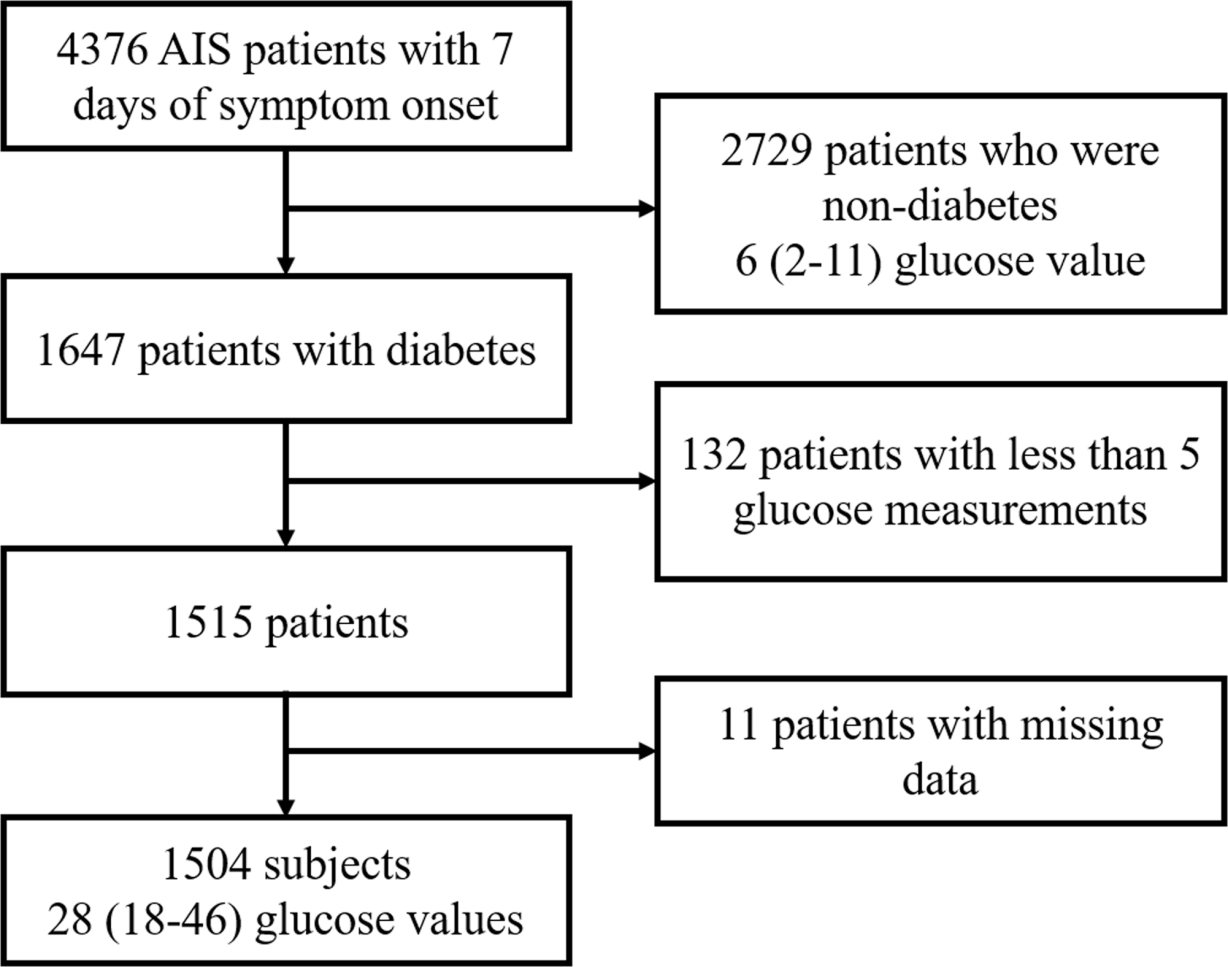
Study participants.

We enrolled patients with AIS from 6 tertiary teaching hospitals who were admitted within 7 days of symptom onset, from January 2013 to December 2014. Of 4,376 patients with AIS, we excluded 2,729 who were not diagnosed with diabetes, as glucose measurements during admission alone were not sufficient to indicate glycemic excursion in the usual clinical settings. Moreover, we excluded patients who underwent glucose measurement less than 5 times during admission (n = 132) and those with missing laboratory data (n = 11) (Fig 1). Written Informed consent was obtained from all the patients or the authorized next of kin, and the institutional review board (IRB) and ethics committee of each participating hospital (IRBs in Hanyang University Hospital, Chuncheon Sacred Heart Hospital, Seoul National University Hospital, Seoul National University Seoul Metropolitan Government Boramae Medical Center, Kyung Hee University Hospital and Seoul National University Bundang Hospital) approved this study.

### Data collection

All the participating centers contributed to the prospective registry of the Clinical Research Center for Stroke; the detailed methods of data collection have been described previously [16]. In brief, this registry includes information on patient demographics and clinical variables and outcomes, in-hospital treatment including thrombolysis, and premorbid conditions including risk factors. Stroke was classified as transient ischemic attack (TIA), large artery atherosclerosis (LAA), small vessel occlusion (SVO), cardioembolic (CE) stroke, stroke of undetermined etiology (SUE), or stroke of other determined etiology (SOE) according to Trial of ORG 10172 in Acute Stroke Trial [17]. The stroke severity at admission was evaluated using the National Institute Health Stroke Scale (NIHSS) score [18]. Hypertension was diagnosed in patients who were taking antihypertensive medications or in patients with an average sitting blood pressure of ≥140 mmHg (systolic) or ≥90 mmHg (diastolic). Diabetes was diagnosed in patients who were receiving medical treatment for diabetes, in those with a fasting serum glucose level of ≥ 126 mg/dL, or those with a non-fasting serum glucose level of ≥ 200 mg/dL, with corresponding symptoms of diabetes, and in those with an admission A1c ≥6.5%. Hyperlipidemia was diagnosed in patients with a total cholesterol level of ≥ 240 mg/dL, or in patients who were treated with lipid lowering agents. Current smoking status was considered in patients who smoked 1 or more cigarettes per day within the last 6 months.

### Blood glucose measurement

All the admitted patients underwent immediate blood glucose measurement to rule out focal neurologic deficits. After the initial assessment, the blood sugar levels were determined 4 times daily (fasting state [7 AM], post-prandial [9 AM], before dinner [4 PM], and at night [10 PM]) by the finger prick method. Cases of hyperglycemia were managed according to the established guidelines, which state that the blood glucose level should be maintained within 140–180 mg/dL. If insulin was injected to maintain the optimal blood glucose level, additional blood tests were permitted to determine whether sufficient blood glucose lowering was achieved or whether the insulin injection induced hypoglycemia. However, blood sugar tests were not performed for all patients after the acute period until an appropriate blood glucose level was achieved. After the acute period of stroke, a blood glucose test was performed if the patients exhibited a detrimental neurologic status or symptomatic hypo- and hyperglycemia. In other cases, the glucose levels were managed as per the current guideline to prevent the development of long-term complications of diabetes [19].

### Glycemic variability index

Capillary glucose levels were obtained sequentially from admission to discharge by using an electronic medical recording system. The mean and median values of glucose during admission were calculated. Hypoglycemia was defined as capillary blood glucose <70 mg/dL. There is no gold standard measurement of glycemic excursion in clinical practice. Most of researchers agree that these standard GV measurement should be more sensitive, reliable, reproducible, and easy to understand [20]. Range of glucose is the one of the simplest and easiest metrics to calculate, and this GV index is associated with the mortality in patients with acute myocardial infarction [21] and with critically illnesses [22]. Therefore, the GV during hospital admission was assessed as range (maximum–minimum) of glucose during the hospital admission. We excluded glucose values less than 10 or more than 600 mg/dL as an outlier.

### Outcome

The functional outcome was assessed by using the modified Rankin Scale (mRS) score [23], which was measured by experienced stroke neurologists or trained nurses during a visit to the outpatient clinic or via a telephone interview. Accordingly, the mRS consists of 7 grades of increasing disability, from 0 (no symptoms) to 6 (death). Poor functional outcome was defined as a dichotomized mRS score of 3–6 at 3 months after stroke onset.

### Statistical analysis

The bivariate correlations among glucose parameters, hypoglycemic episodes, and crude mRS scores were examined using a Spearman’s rank correlation test. Baseline contingency table was generated to determine the differences in the demographic and clinical characteristics based on range of glucose 4 quartiles. Continuous variables are expressed as mean ± SD or median (interquartile range, IQR), whereas categorical variables are expressed as number (%). The differences in the baseline characteristics according to the blood glucose range quartiles were examined using analysis of variance, with the Bonferroni post-hoc test and the Kruskal-Wallis test for continuous variables, and with the Pearson’s chi-square test for categorical variables. Univariable analyses of the predictors of poor functional outcome (dichotomized mRS 3–6) at 3 months after stroke onset were used to determine the unadjusted associations between the dependent variables, including age, gender, stroke risk factors (prior history of stroke, hypertension, hyperlipidemia, and current smoking), clinical characteristics (stroke subtype, stroke severity, IV thrombolysis), and glucose parameters (number of glucose measurement, A1c level, hypoglycemic event, and range of glucose quartile). The NIHSS score was an interval variable with a non-normal distribution, and was hence divided into 4 groups: 0–1, 2–3, 4–6, and ≥7. We used the glucose range quartile as the dependent variable to estimate the odds ratios (ORs) for poor functional outcome at 3 months after stroke onset. Multivariable binary logistic regression analyses were performed using a tiered approach. First, the demographic variables (age and sex) were added into the model. Second, the risk factors and characteristics of stroke (thrombolysis, stroke severity, and subtype) were added to the model. The number of glucose measurement and hypoglycemic event were included in the final model. In addition, mRS score 5 and 6 were combined with one variable in multivariable ordinal logistic regression analysis. Covariates with a variance inflation factor of >3 were removed from the model to minimize the multicollinearity. Statistical significance was set at *P* <0.05, and all statistical analyses were performed with the IBM SPSS 21.0 software.

## Results

### Baseline characteristics

Among the 1,504 diabetic patients with AIS, the mean and median age was 68.1±10.7 and 70.0 (interquartile range, 61.0–76.0) years, respectively. And, the proportion of male patients was 57.2% (Table 1). Over a median period of 7.1 (range, 4.7–12.0) days of admission, a total of 57,584 capillary glucose measurements were sequentially conducted 28 (range, 18–46) times in each patient. The measured capillary glucose values ranged from 15 to 587 mg/dL, and the mean glucose value was 169±35 mg/dL. The mean A1c value was 7.4±1.7%. Hypoglycemia was noted in 222 of 1,504 subjects (14.8%). LAA was the predominant stroke subtype in the study population. Compared to patients with a lower glucose range quartile, those with a higher quartile more frequently had a history of hypertension and hyperlipidemia. The stroke severity proportionally increased with an increase in the glucose range quartile (*P* <0.001, Kruskal-Wallis test). In addition, 96 patients (6.4%) received thrombolysis, and the proportion of patients who received thrombolysis did not differ according to the glucose range quartile. The levels of glucose and A1c at admission, as well as the mean, median and maximum values of the glucose parameters during admission proportionally increased in cases from the lowest to highest glucose range quartile. In particular, the minimum value of the glucose parameters decreased and the proportion of cases with hypoglycemia increased as the glucose range quartile increased. Furthermore, the glucose range significantly differed (*P* <0.001, Kruskal-Wallis test) according to the anti-diabetic treatment modality, with the lowest values in the no treatment group and the highest values in the insulin group (S1 Fig).

**Table 1:** Baseline characteristics.

	Range of glucose					*P* value
	Total (N = 1,504)	Quartile 1 (0–129 mg/dL, N = 383)	Quartile 2 (130–175 mg/dL, N = 378)	Quartile 3 (176–235 mg/dL, N = 371)	Quartile 4 (>235 mg/dL, N = 372)	
Age, years	68.1±10.7	68.6±11.1	67.8±10.4	68.3±10.8	67.8±10.6	0.375
Male, %	860 (57.2)	205 (53.5)	232 (61.4)	220 (59.3)	203 (54.6)	0.089
Risk factors, %						
Previous stroke	393 (26.1)	91 (23.8)	96 (25.4)	95 (25.6)	111 (29.8)	0.270
Hypertension	1,114 (78.1)	315 (82.2)	301 (79.6)	277 (74.7)	281 (75.5)	0.039
Hyperlipidemia	648 (43.1)	187 (48.8)	171 (45.2)	159 (42.9)	131 (35.2)	0.002
Current smoking	376 (25.0)	92 (24.0)	90 (23.8)	107 (28.8)	87 (23.4)	0.271
Atrial fibrillation	210 (14.0)	53 (13.8)	55 (14.6)	41 (11.1)	61 (16.4)	0.206
TIA and TOAST subtype, %						0.199
TIA	56 (3.7)	21 (5.5)	13 (3.4)	13 (3.5)	9 (2.4)	
LAA	534 (35.5)	133 (34.7)	119 (31.5)	143 (38.5)	139 (37.4)	
SVO	428 (28.5)	101 (26.4)	124 (32.8)	110 (29.6)	93 (25.0)	
CE	269 (17.9)	66 (17.2)	71 (18.8)	63 (17.0)	69 (18.5)	
SOE	24 (1.6)	5 (1.3)	7 (1.9)	5 (1.3)	7 (1.9)	
SUE	193 (12.8)	57 (14.9)	44 (11.6)	37 (10.0)	55 (14.8)	
NIHSS score	3 (1, 6)	2 (1, 4)	3 (1, 6)	3 (1, 6)	4 (2, 8)	<0.001
IV Thrombolysis, %	6.4	6	5	6.7	7.8	0.462
Glucose parameters						
Number of measurements	28 (18–46)	19 (13–25)	27 (18–38)	31 (22–50)	45 (30–73)	<0.001
Mean, mg/dL	169±35	142±23	162±26	175±28	199±35	<0.001
Median, mg/dL	163±37	137±24	156±29	169±30	191±39	<0.001
Maximum, mg/dL	283±80	199±31	251±25	297±30	388±61	<0.001
Minimum, mg/dL	95±25	102±20	100±21	95±26	84±29	<0.001
at admission, mg/dL	182±76	145±40	167±55	183±63	235±103	<0.001
A1c, %	7.4±1.7	6.7±1.1	7.2±1.4	7.6±1.6	8.2±2.1	<0.001
Hypoglycemic event, %	14.8	3.1	5.8	17	33.6	<0.001
Length of hospital stay, days	7.2 (4.7–12.1)	5.3 (3.5–7.8)	6.8 (4.5–10.3)	7.2 (4.9–12.6)	10.6 (6.9–17.8)	<0.001

TIA, transient ischemic attack; TOAST, Trial of Org 10172 in Acute Stroke Treatment; LAA, Large Artery Atherosclerosis; SVO, Small Vessel Occlusion; CE, Cardioembolism; SOE, Stroke of Other determined Etiology; SUE, Stroke of Undetermined Etiology; NIHSS, National Institutes of Health Stroke Scale; IV, Intravenous; A1c, glycated hemoglobin.

### Glucose parameters, crude mRS, and hypoglycemic event

All the participants had a median mRS score of 2 (range, 1–3) at 3 months after stroke onset. The glucose value at admission was not correlated with a crude mRS score or hypoglycemic event in our population (Table 2). Range of glucose was correlated to a significantly greater extent with the crude mRS score (*r* = 0.201, *P* <0.001) and hypoglycemic event (*r* = 0.337, *P* <0.001).

**Table 2:** Matrix of correlation coefficient among crude modified Rankin Scale score, hypoglycemia rate and glucose parameters.

Characteristics	(1)	(2)	(3)	(4)	(5)	(6)	(7)	(8)	(9)	(10)
(1) Number of glucose measurements	–									
(2) Glucose at admission	0.098^a^	–								
(3) Mean	0.167^a^	0.510^a^	–							
(4) Median	0.154^a^	0.481^a^	0.968^a^	–						
(5) Maximum	0.421^a^	0.474^a^	0.787^a^	0.704^a^	–					
(6) Minimum	0.351^a^	0.172^a^	0.375^a^	0.364^a^	0.006	–				
(7) Range	0.520^a^	0.389^a^	0.624^a^	0.551^a^	0.944^a^	0.238^a^	–			
(8) A1c	0.059^a^	0.375^a^	0.460^a^	0.439^a^	0.399^a^	0.148^a^	0.331^a^	–		
(8) mRS	0.375^a^	-0.011	0.103^a^	0.100^a^	0.177^a^	0.124^a^	0.201^a^	0.046	–	
(9) Hypoglycemia, %	0.283^a^	0.026	0.066^a^	0.069^a^	0.170^a^	0.614^a^	0.337^a^	0.034	0.138^a^	–

A1c, glycated hemoglobin; mRS, modified Rankin Scale score

Values are presented with Spearman’s correlation coefficient.

^a^*P* value <0.05

### Predictors of poor outcome

A total of 528 subjects (35.1%) had a poor functional outcome at 3 months after stroke onset. In the univariable analysis, the mean glucose level (OR, 1.01; 95% confidence interval [CI], 1.01–1.01; *P* = 0.001) and hypoglycemic event (OR, 2.26; 95% CI: 1.70–3.02; *P* < 0.001) were associated with poor outcomes (Table 3). However, the A1c level was not associated with poor outcomes. Each glucose range quartile, relative to the first quartile, was positively associated with increasing ORs for poor outcome at 3 months after stroke onset, according to the univariable analysis (*P* for chi-square trend <0.001).

**Table 3:** Univariable analyses of the predictors of poor functional outcome at 3 months after stroke onset.

	Favorable outcome (N = 976)	Poor outcome (N = 528)	Univariable OR (95% CI)	*P* value
Age, years	66.5±10.6	71.1±10.4	1.04 (1.03–1.06)	<0.001
Gender, male (%)	60.5	51.1	0.67 (0.55–0.85)	0.001
Risk factor, %				
Previous stroke	20.2	37.1	2.33 (1.84–2.96)	<0.001
Hypertension	76.9	80.1	1.21 (0.93–1.57)	0.157
Hyperlipidemia	42.6	43.9	1.06 (0.85–1.31)	0.623
Current smoking	28.2	19.1	0.60 (0.47–0.78)	<0.001
TIA and TOAST subtype, %				0.008
TIA	4.7	1.9	0.49 (0.24–1.00)	0.049
LAA	34.5	37.3	1.31 (1.00–1.72)	0.050
SVO	30.3	25.0	1.0 (reference)	-
CE	16.7	20.1	1.46 (1.06–2.01)	0.021
SOE	1.7	1.3	0.92 (0.37–2.28)	0.863
SUE	12.0	14.4	1.46 (1.02–2.08)	0.037
NIHSS score, %				
0–1	37.0	11.0	1.0 (reference)	-
2–3	30.4	20.1	2.22 (1.56–3.17)	<0.001
4–6	22.3	25.2	3.80 (2.67–5.40)	<0.001
≥7	10.2	43.8	14.38 (10.00–20.67)	<0.001
IV thrombolysis, %	4.8	9.3	2.02 (1.34–3.06)	0.001
Glucose parameters				
Mean, mg/dL	167±34	173±36	1.01 (1.00–1.01)	0.001
Hypoglycemia rate, %	11.0	21.8	2.26 (1.70–3.02)	<0.001
A1c, %	7.4±1.7	7.4±1.7	1.00 (0.94–1.07)	0.947
Range quartile, %				
Q1	37.0	11.0	1.0 (reference)	-
Q2	30.4	20.1	1.66 (1.20–2.29)	0.002
Q3	22.3	20.1	2.44 (1.78–3.36)	<0.001
Q4	10.2	43.8	3.09 (2.26–4.25)	<0.001

TIA, transient ischemic attack; TOAST, Trial of Org 10172 in Acute Stroke Treatment; LAA, Large Artery Atherosclerosis; SVO, Small Vessel Occlusion; CE, Cardioembolism; SOE, Stroke of Other determined Etiology; SUE, Stroke of Undetermined Etiology; NIHSS, National Institutes of Health Stroke Scale; IV, Intravenous; A1c, glycated hemoglobin; Q1-4, quartile 1–4; CI, confidence interval.

In the multivariable binary logistic regression analysis (Table 4), the glucose range quartile remained significant, after adjusting for age, gender, NIHSS quartile, IV thrombolysis, stroke subtype, number of glucose measurement, and hypoglycemic event (OR for quartile 2 vs. 1: 1.50, 95% CI: 1.02–2.18; OR for quartile 3 vs. 1: 2.01, 95% CI: 1.34–3.01; OR for quartile 4 vs. 1: 1.98, 95% CI: 1.22–3.23). In addition, range of glucose quartile were positively associated with increased risk of unfavorable shift in mRS score distribution (adjusted common OR for quartile 2 vs. 1: 1.31, 95% CI: 0.99–1.74; OR for quartile 3 vs. 1: 1.72, 95% CI: 1.30–2.28; OR for quartile 4 vs. 1: 2.46, 95% CI: 1.82–3.33; S1 Table).

**Table 4:** Multivariable binary logistic regression analyses of the predictors of poor functional outcomes at 3 months after stroke onset, according to the range quartile.

	Odds ratio (95% CI)			
	Range Q1	Range Q2	Range Q3	Range Q4
Crude	Reference	1.66 (1.20–2.29)	2.44 (1.78–3.36)	3.09 (2.26–4.25)
Age- and sex-adjusted	Reference	1.79 (1.29–2.50)	2.63 (1.90–3.65)	3.43 (2.47–4.74)
Model 1	Reference	1.79 (1.28–2.51)	2.67 (1.91–3.72)	3.41 (2.45–4.75)
Model 2	Reference	1.58 (1.10–2.27)	2.34 (1.64–3.35)	2.69 (1.88–3.86)
Model 3	Reference	1.60 (1.11–2.31)	2.36 (1.65–3.38)	2.69 (1.88–3.87)
Model 4	Reference	1.44 (1.00–2.08)	1.93 (1.33–2.79)	1.87 (1.26–2.78)
Model 5	Reference	1.44 (1.00–2.08)	1.86 (1.28–2.70)	1.72 (1.15–2.59)

CI, confidence interval; Q1–4, quartile 1–4.; CI, confidence interval.

Model 1: adjusted for history of stroke, hypertension, hyperlipidemia, and current smoking

Model 2: Model 1 plus adjustment for NIHSS score quartile and intravenous thrombolysis

Model 3: Model 2 plus adjustment for TOAST classification

Model 4: Model 3 plus adjustment for number of glucose measurements

Model 5: Model 4 plus adjustment for hypoglycemia.

**Fig 2:**
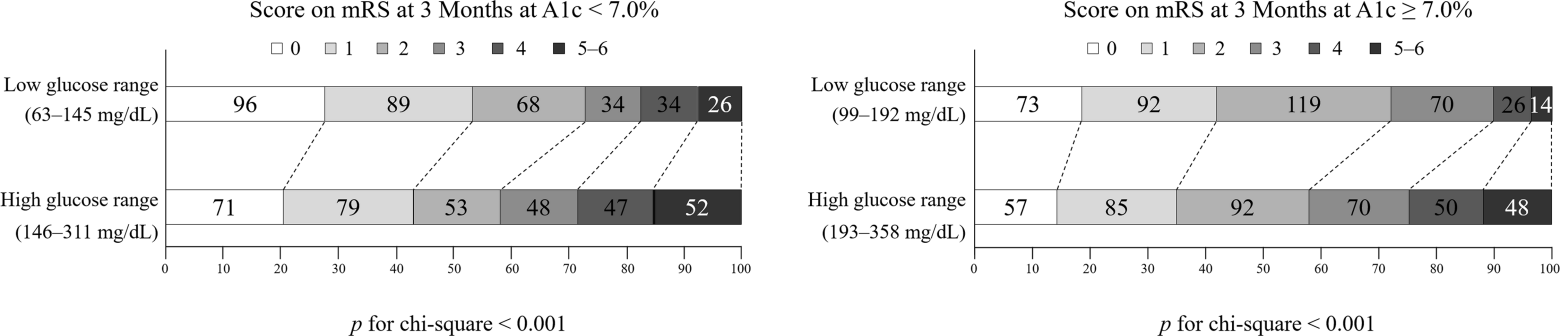
Distribution of the modified Rankin Scale (mRS) scores at 3 months after stroke onset according to the glucose range and glycated hemoglobin (A1c) level.

We performed subgroup analysis wherein the glucose range was divided as high and low based on the A1c values to compare the effect of the previous diabetic control status on the functional outcome. Patients with a lower glucose range had a better outcome irrespective of the A1c level at admission, as compared to those with a higher glucose range (Fig 2).

**Fig 3:**
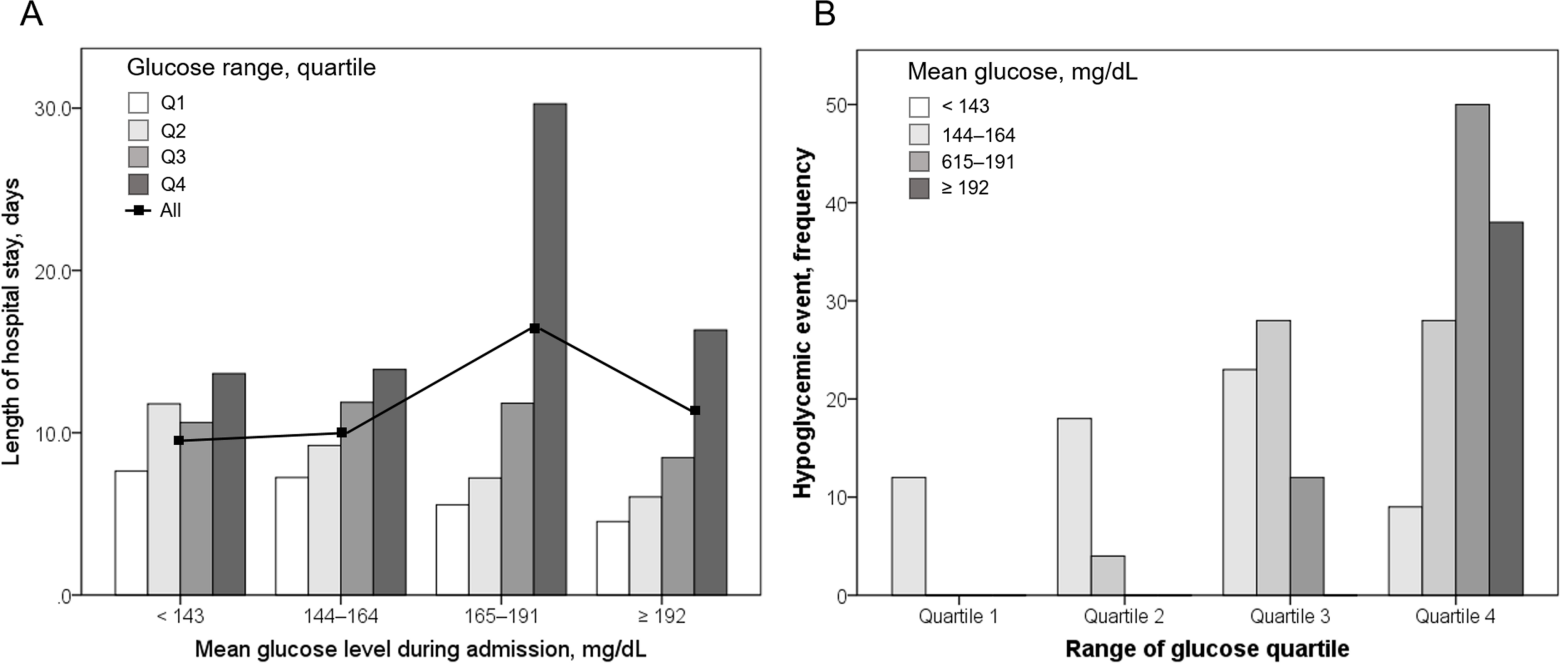
Relationship between length of hospital stay (A), frequency of hypoglycemic event (B) and glucose parameters during admission.

Relationships between length of hospital stay, frequency of hypoglycemic event and glucose parameters are summarized in Fig 3. Length of hospital stay (Fig 3A) and frequency of hypoglycemic event (Fig 3B) was gradually increased as the quartiles of glucose range moved toward the highest quartile. However, length of hospital stay was not significantly different according to mean glucose level. Increased quartiles of mean glucose level was significantly associated with a higher rate of hypoglycemic event.

## Discussion

In this retrospective observational study, we found that a high GV—represented by glucose range quartiles—was associated with poor functional outcome at 3 months after stroke onset in patients with diabetes and AIS. This association remained significant after adjusting for number of glucose measurement and the hypoglycemic event. Range of glucose may be useful to evaluate the association between glycemic excursion and functional outcome in patients with AIS in actual clinical settings.

The association between GV and poor outcome in patients with AIS can be explained from various viewpoints. First, an increased GV would indicate poor control of diabetes. In Table 1, higher glucose range quartiles, relative to a low quartile, were associated with increased glucose and A1c values at admission. Therefore, GV could serve as a marker for poor control of diabetes prior to stroke [24,25]. Second, hyperglycemia at admission has been reported to occur as a result of stress response after stroke, wherein hyperglycemia is associated with an increased cortisol level [26]. As the severity and lesion size of ischemic stroke increase, the serum cortisol level would also proportionally increase [27–29], which could consequently result in elevated glucose levels at admission. We found that the initial stroke severity was significantly associated with an increased GV. This could be partially explained by the fact that high GV reflects the severity of ischemic stroke. Third, several studies have shown that increased GV could predict hypoglycemic episodes in patients with type 2 diabetes [29,30]. Hypoglycemia is a significant predictor of worse outcomes in various clinical settings [31,32]. In our study, the proportion of patients with hypoglycemia increased with an increase in the glucose quartile range (Fig 3B). An increase in GV is known to be a marker for hypoglycemia [33,34]. Thus, an increased glucose range could affect stroke outcomes via harmful impacts of overt or covert hypoglycemia. In our data, length of hospital stay and frequency of hypoglycemic event was not associated with an increased level of mean glucose level. Instead, increased frequency of hypoglycemic event were associated with an increased quartiles of range of glucose. Hypoglycemia may be developed when physicians normalize the high level of glucose in acute clinical setting. We could not be convinced whether hypoglycemia has a deleterious effect on stroke outcome. However, hypoglycemia should be prevented or reduced whenever possible because range of glucose are positively associated with the development of hypoglycemia during hospital admission.

Intensive insulin therapy in patients with acute stroke failed to show of its efficacy in functional outcome and mortality [35]. In our data, increased glycemic variability (range of glucose) was associated with a higher usage of insulin treatment and a higher incidence of hypoglycemia during admission (Fig 3B and S1 Fig). The effort to correct hyperglycemia as well as to reduce glycemic variability could be lessen the effect of glucose normalization in acute stroke trials because of the harmful effect of hypoglycemic event.

In our data, there were apparently higher proportion of patients with hypertension and dyslipidemia in lower glucose quartile range groups compared to those with higher glucose quartile range group. Though we did not assess medication adherence of our participants, there is a possibility for the high medication adherence in patients with higher glucose range quartile than in those with lower quartile. In Table 1, patients with lower glucose range quartile showed low admission glucose and A1c levels. These findings suggested that their medication adherence might be higher than those with lower glucose range quartile, because medication adherence was positively associated with the presence of multiple cardiovascular risk factors [36].

There were no “gold standard” for the assessment of glycemic excursion in patients with diabetes and stroke [37]. It is unclear why the glucose range quartile during hospitalization is associated to a greater extent with functional outcomes in AIS patients, as compared to the GV indices, such as standard deviation (SD) or coefficient of variation (CV) of glucose values. Allport et al studied the temporal profile of glucose levels in patients with AIS via continuous glucose monitoring, and found that the glucose values fluctuated to a greater extent during the early period after AIS than during the late period [38]. Hence, it is possible that the degree of GV (including the SD or CV of the glucose values) could be minimized, as the measured glucose values included a greater number of values from the hyperacute period through the entire hospital admission period. In another study, SD, CV, or mean absolute change of glucose levels during the acute period were associated with mortality or poor prognosis in critically ill patients [39,40]. Accordingly, we suggested that the SD or CV of glucose values are more suitable for evaluating GV in the hyperacute period, as compared to the glucose range. However, as frequent glucose sampling during the hyperacute period was not recommended in our practice guidelines, it is difficult to obtain the SD or CV of the glucose values only in the hyperacute period after stroke.

We collected all capillary glucose data during hospital admission. Several studies suggested that glucose excursions in the acute period are more important than those in the chronic period, when evaluating clinical outcomes [41,42]. However, post-stroke hyperglycemia could develop more than 48 hours after symptom onset [38]. Hypoglycemia may develop during the chronic period as iatrogenic hypoglycemia due to the persistent use of anti-diabetic treatment or the relative paucity of glucose monitoring during this period. The glucose range reflects the fluctuations in the entire glucose profile in a simple manner. Moreover, the glucose range is easier to calculate and use to make clinical decisions, as compared to the SD or CV of the glucose values. In fact, a reduction in the glucose range by 10 points is more intuitive as compared to a reduction of 10% in the SD or CV of the glucose values. In this perspectives, reducing range of glucose during hospital admission might be effective management strategy in reducing the development of hypoglycemia and in finally improving functional outcomes in patients with an acute ischemic stroke.

Our study has several limitations. First, we could not show a causal relationship between GV and functional outcome in patients with AIS due to the retrospective nature of our observations. Second, we could not consider the differential effect of nutritional status on GV and outcomes. Although continuous glucose monitoring data showed that glycemic profiles did not differ according to feeding status or diabetes status [39], the relationship between GV and nutritional status should be investigated in future studies, as nutritional status is associated with the development of hypoglycemia. Third, the exclusion of some patients (for example, due to the unavailability of glucose measurement data) could weaken the generalizability of our data in diabetic patients with AIS.

## Conclusions

In conclusion, we found that the glucose range, as a GV index, is positively associated with poor functional outcomes at 3 months after stroke onset in diabetic patients with AIS. This harmful association remained significant irrespective of number of glucose measurement or hypoglycemic event, and the glucose range remained a significant predictor of worse outcome. Future prospective study should assess whether GV improvement after AIS can lead to improved functional outcomes.

## Supporting information

S1 Fig Results of the Kruskal-Wallis test showing the glucose range against the types of anti-diabetic treatment. OHA, oral hypoglycemic agent.
(TIF)

S1 Table Multivariable ordinal logistic regression analyses of the predictors of unfavorable shift in modified Rankin Scale score distribution at 3 month. OR, odds ratio; CI, confidence interval; TIA, transient ischemic attack; TOAST, Trial of Org 10172 in Acute Stroke Treatment; LAA, Large Artery Atherosclerosis; SVO, Small Vessel Occlusion; CE, Cardioembolism; SOE, Stroke of Other determined Etiology; SUE, Stroke of Undetermined Etiology; NIHSS, National Institutes of Health Stroke Scale; IV, Intravenous; A1c, glycated hemoglobin; Q1–4, quartile 1–4.
(DOCX)
