# Supplementary figures and images for: Range of glucose as a glycemic variability and 3–month outcome in diabetic patients with acute ischemic stroke
Source: PLoS One. 2017 Sep 7;12(9):e0183894. doi: 10.1371/journal.pone.0183894 (PMC5589173; doi:10.1371/journal.pone.0183894)

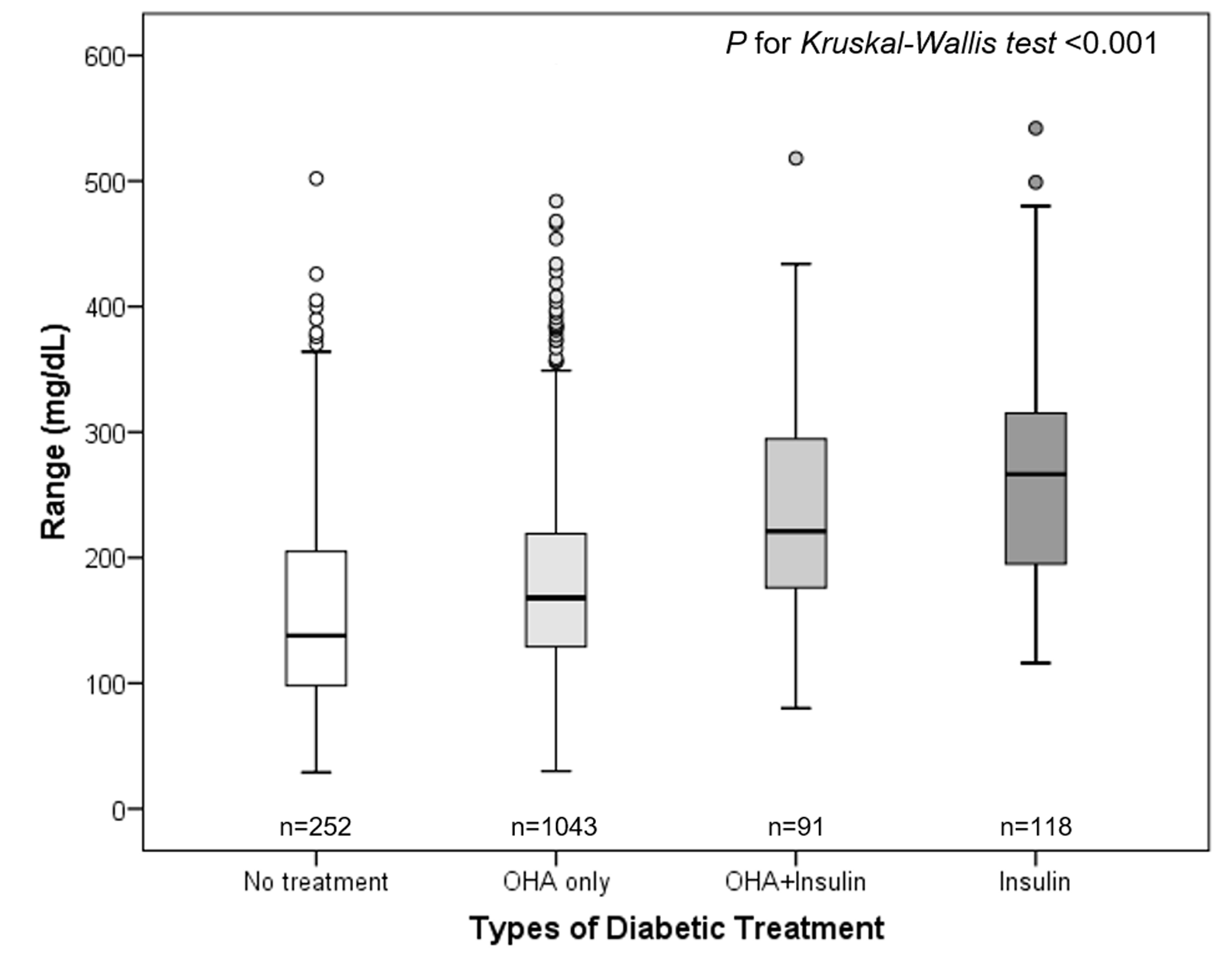

Supplement: S1 Fig — OHA, oral hypoglycemic agent. (TIF) [file pone.0183894.s001.tif]
