# Supplementary material for: Range of glucose as a glycemic variability and 3–month outcome in diabetic patients with acute ischemic stroke
Source: PLoS One. 2017 Sep 7;12(9):e0183894. doi: 10.1371/journal.pone.0183894 (PMC5589173; doi:10.1371/journal.pone.0183894)
Supplement: S1 Table — OR, odds ratio; CI, confidence interval; TIA, transient ischemic attack; TOAST, Trial of Org 10172 in Acute Stroke Treatment; LAA, Large Artery Atherosclerosis; SVO, Small Vessel Occlusion; CE, Cardioembolism; SOE, Stroke of Other determined Etiology; SUE, Stroke of Undetermined Etiology; NIHSS, National Institutes of Health Stroke Scale; IV, Intravenous; A1c, glycated hemoglobin; Q1–4, quartile 1–4. (DOCX) [file pone.0183894.s002.docx]

**S1 Table. Multivariable ordinal logistic regression analyses of the predictors of unfavorable shift in modified Rankin Scale score distribution at 3 month.**

|  | Univariable OR (95% CI) |  | Multivariable OR (95% CI) |
| --- | --- | --- | --- |
| Age, years | 1.04 (1.03–1.05) |  | 1.04 (1.03–1.05) |
| Gender, male | 0.76 (0.63–0.90) |  | 0.90 (0.74–1.11) |
| Risk factor |  |  |  |
| Previous stroke | 2.17 (1.77–2.68) |  | 1.67 (1.33–2.10) |
| Hypertension | 1.24 (1.00–1.53) |  | – |
| Hyperlipidemia | 1.06 (0.89–1.27) |  | – |
| Current smoking | 0.72 (0.59–0.88) |  | 1.08 (0.83–1.40) |
| TIA & TOAST subtype |  |  |  |
| TIA | 0.30 (0.17–0.50) |  | 0.33 (0.17–0.63) |
| LAA | 1.29 (1.04–1.62) |  | 0.94 (0.73–1.20) |
| SVO | 1.0 (reference) |  | 1.0 (reference) |
| CE | 1.35 (1.03–1.77) |  | 0.76 (0.56–1.04) |
| SOE | 0.88 (0.41–1.89) |  | 1.32 (0.56–3.12) |
| SUE | 1.36 (1.01–1.83) |  | 1.00 (0.72–1.38) |
| NIHSS scale |  |  |  |
| 0–1 | 1.0 (reference) |  | 1.0 (reference) |
| 2–4 | 2.34 (1.85–2.97) |  | 2.14 (1.68–2.72) |
| ≥ 5 | 9.06 (6.91–11.90) |  | 8.31 (6.21–11.14) |
| IV thrombolysis | 1.91 (1.32–2.76) |  | 0.63 (0.42–0.95) |
| Glucose parameters | – |  | – |
| Mean | 1.01 (1.00–1.01) |  | – |
| Hypoglycemic event | 2.05 (1.59–2.63) |  | 1.14 (0.85–1.53) |
| A1c | 1.00 (1.00–1.00) |  | – |
| Range quartile |  |  |  |
| Q1 | 1.0 (reference) |  | 1.0 (reference) |
| Q2 | 1.51 (1.18–1.94) |  | 1.31 (0.99–1.74) |
| Q3 | 1.96 (1.52–2.52) |  | 1.72 (1.30–2.28) |
| Q4 | 2.82 (2.17–3.65) |  | 2.46 (1.82–3.33) |

OR, odds ratio; CI, confidence interval; TIA, transient ischemic attack; TOAST, Trial of Org 10172 in Acute Stroke Treatment; LAA, Large Artery Atherosclerosis; SVO, Small Vessel Occlusion; CE, Cardioembolism; SOE, Stroke of Other determined Etiology; SUE, Stroke of Undetermined Etiology; NIHSS, National Institutes of Health Stroke Scale; IV, Intravenous; A1c, glycated hemoglobin; Q1–4, quartile 1–4.
